# Time-to-Boundary Function to Study the Development of Upright Stance Control in Children

**DOI:** 10.2174/1874120701711010049

**Published:** 2017-04-28

**Authors:** Carmen D'Anna, Maurizio Schmid, Andrea Scorza, Salvatore A. Sciuto, Luisa Lopez, Silvia Conforto

**Affiliations:** 1 Department of Engineering, Università degli Studi Roma Tre, Rome, Italy; 2 Rehabilitation Center for Developmental Disorders, Villaggio Eugenio Litta, Grottaferrata, Rome, Italy

## Abstract

**Background:**

The development of postural control across the primary school time horizon is a complex process, which entails biomechanics modifications, the maturation of cognitive ability and sensorimotor organization, and the emergence of anticipatory behaviour. Postural stability in upright stance has been thus object of a multiplicity of studies to better characterize postural control in this age span, with a variety of methodological approaches. The analysis of the Time-to-Boundary function (TtB), which specifies the spatiotemporal proximity of the Centre of Pressure (CoP) to the stability boundaries in the regulation of posture in upright stance, is among the techniques used to better characterize postural stability in adults, but, as of now, it has not yet been introduced in developmental studies. The aim of this study was thus to apply this technique to evaluate the development of postural control in a sample population of primary school children.

**Methods:**

In this cross-sectional study, upright stance trials under eyes open and eyes closed were administered to 107 healthy children, divided into three age groups (41 for Seven Years' Group, Y7; 38 for Nine Years' Group, Y9; 28 for Eleven Years' Group, Y11). CoP data were recorded to calculate the Time-to-Boundary function (TtB), from which four spatio-temporal parameters were extracted: the mean value and the standard deviation of TtB minima (M_min_, Std_min_), and the mean value and the standard deviation of the temporal distance between two successive minima (M_dist_, Std_dist_).

**Results:**

With eyes closed, M_min_ and Std_min_ significantly decreased and M_dist_ and Std_dist_ increased for the Y7 group, at Y9 M_min_ significantly decreased and Std_dist_ increased, while no effect of vision resulted for Y11. Regarding age groups, M_min_ was significantly higher for Y9 than Y7, and Std_min_ for Y9 was higher than both Y7 and Y11; M_dist_ and Std_dist_ resulted higher for Y11 than for Y9.

**Conclusion:**

From the combined results from the spatio-temporal TtB parameters, it is suggested that, at 9 years, children look more efficient in terms of exploring their limits of stability than at 7, and at 11 the observed TtB behaviour hints at the possibility that, at that age, they have almost completed the maturation of postural control in upright stance, also in terms of integration of the spatio-temporal information.

## 1 INTRODUCTION

The postural control is the result of a long-lasting development process, closely linked to the neuro-development of different mechanisms related respectively to the central nervous system (CNS), the motor abilities and the sensory channels. This development is very complex because its main steps occur at different ages: the CNS responses and its changes occur in the first years of life; the somatosensory system matures first, followed by the visual and then vestibular system; the integration of all sensory systems occurs between the ages of 4 and 6 years. Developmental studies involving postural control suggest that anticipatory control, despite its early emergence, slowly matures during childhood reflecting the maturation of the CNS.

Therefore, the study of the postural control development has been a matter of research for many years [1, 2]. The major question was to understand when the development of the system could be considered as completed and what are the factors influencing it. Then, the development of postural control has been studied from different points of view such as the neurophysiological one, through the development of theories and models, and the biomechanical one through the analysis of the variations of sway occurring when the age increases.

In 1991, Ashmead and McCarty [3] showed that infants of 12-14 months, who could stand independently, swayed more than adults in difficult standing. Barela *et al.* (2000) [4] showed that the body sway in infants started to decrease after some experience in walking without support, so highlighting the close link between the postural development and the acquisition of the motor function. Some postural control studies focusing on adjustments during standing and walking showed that, after infancy, children enter a transitional phase at 5-6 years [5]. It has been hypothesized that prior to this age, the postural sway is controlled by an open-loop strategy and relies more on somatosensory than on visual information. Kirschenbaum *at al.* (2001) have shown, furthermore, that the postural control development is not linearly related with age [6] and that it follows the maturation of fine competencies in muscular coordination [7]. Even if it is known that the body sway decreases with age [8-10], some conflicting opinions still debate about the age at which children exhibit signs of an adult-like postural control strategy, that is when the “transitional” period ends. It was showed that this “transitional” period could depend on different developmental processes including the complete development of the visual acuity, the achievement of the maximum efficiency of the vestibular system and the completion of the anticipatory postural behavior, characterized by an active feed-forward control.

Riach *et al.* (1994) examined cross-sectionally the characteristics of postural sway in healthy children of different ages, by studying the spectral composition of sway, and highlighted that children, until the age of 7 years, use visual information differently from adults [9]. Taguchi *et al.* (1988) reported that the amplitude of spontaneous postural sway in children aged 9-12 with eyes open was comparable to that of adults in the same conditions [10]. Peterson *et al.* (2006) suggested that children do not exhibit an adult-like sensory information use prior to age of 12 years [11].

The study conducted by Ferronato *et al.* (2011) aimed at identifying and quantifying the two components of the Centre of Pressure (CoP) – rambling (the migration of the reference point) and trembling (the deviation away from the reference point) [12] – has shown no difference with respect to the adults after the age of 8 years, even though the overall CoP displacement still appeared larger than in adults [13].

Schmid *et al.* (2005) investigated the variations and the development of balance control mechanisms in children from 7 to 11, considering two different visual conditions (eyes open and eyes closed), through the analysis of the classical measures extracted directly from the Centre of Pressure (CoP). They showed that the traditional posturographic parameters are sensitive to the vision condition, confirming the thesis that the visual input contribution plays a role that is relevant and that varies with the age. They also suggested that the postural control does not develop monotonically and that it is not yet complete even at the age of 11 [14].

The important role of the vision in postural development is not a novel finding but it has been outlined even in old studies that reported some evidences in that sense.

Among those studies, one of the oldest ones was conducted by Forssberg and Nashenr (1982) in children aged 1½ -10 years. In this paper, the authors showed a pair of very important elements such as: i) either the absence or the impairment of vision minimally affect the postural sway; ii) the conflict between visual and somatosensory information, in children younger than 7 years, produces inappropriate postural adjustments and in some cases the loss of balance [15].

Some years later, Slobounouv and Newell (1994) addressed the effect of the eye closure on the sway area in 3 and 5 years old children; they showed that at 3 years the area was larger than at 5 years and that the eye closure resulted in a reduced sway especially in the 3 year old [16].

An improvement of the visual control consisting in the integration of vision with sensory information appears at around 7-8 years [15], even if until 14 years the children do not replicate the visual or vestibular control of the adults [17]. Further evidences about the turning point – in terms of age – of the visual integration have been proposed by Portfors-Yeomans and Riach (1995) that, by analyzing the frequency characteristics of postural signals, outlined how for children aged from 4 to 6 years, unlike older children and adults, the closure of the eyes does not induce an increased sway. In such a way, the authors hypothesized that young children do not use vision to control posture and change the control strategy at around 7 years (from an open-loop control with fast high-frequency corrections to a slower closed-loop control) [18].

Dealing with the quantitative assessment of balance control, some standard measures, extracted from the CoP, have been typically used. However, since it has been proven that the traditional spatial measures provide limited information regarding the overall postural stability and its development [19], in order to overcome this limitation some new proposals have been provided and their advantages and limitations have been analysed. Among those the postural Time-to Boundary function (TtB) has been demonstrated to detect new elements of the postural control that are often hidden in the traditional measures [20]. However, up to now this function has not been tested in studies dealing with postural control development.

TtB incorporates both spatial and temporal aspects of postural sway [21] and borrows the theory of the time-to-collision from visual perception studies into the movement dynamics of upright stance trials: it uses current position, velocity and acceleration of the CoP to estimate the time required for the CoP coordinates to travel along the trajectory and reach the boundaries of the area of stability. This predictive variable is directly perceivable by the individual and provides information regarding the time needed to reverse a perturbation before loss of balance [22].

The TtB function has been shown to follow a pseudo-periodic behaviour, with the alternation of valleys (minima), when approaching the boundary limits, and peaks (maxima), when turning from one direction to another. The average value of the TtB minima and its standard deviation are two of the parameters extracted from the function: the first is associated with the biomechanical constraints; the second depends on the shape of the CoP trajectory with respect to the boundary limits. The information about the temporal distance between successive minima (mean value and standard deviation) is, instead, representative of the intervention rate of the postural control: the inversion of the TtB function is a direct consequence of the ability of the control system to move the CoP away from the limits of stability [23, 24]: correspondingly, a lower average value of this temporal distance can be hypothesized as linked to a huger intervention rate of the control system.

Previous research has suggested that TtB is more sensitive and effective than traditional parameters in studying postural control [25] in different adult population samples (i.e young, old people) [26, 27], both in healthy participants and in presence of musculoskeletal disorders [28]. It has been used to asses postural control in blind children population [29] and it has been used to provide participants with a visual biofeedback in upright stance [30, 31].

Recently, the parameters extracted from TtB were used to detect postural deficits that traditional parameters were unable to detect, in particular for unilateral chronic ankle instability [28] and for anterior knee pain [32].

However, no study has evaluated yet the development of postural control in children population using the TtB function. Therefore, the goal of this study is to evaluate if these measures can provide additional information about postural control development in children population.

## 2 MATERIALS AND METHODS

The sample population and the experimental protocol refers to the study [14] and it is summarized following.

One-hundred and seven children were selected from classes of three different grades in one primary school, after obtaining proper informed consent from parents and teachers to participate in the study. None of the children had educational needs or certified disabilities. They were divided into three age groups (n= 41 for Seven Years' Group, Y7, n = 38 for Nine Years' Group, Y9, and n = 28 for Eleven Years' Group, Y11). The population anthropometric data are reported in Table **1**.

The participants stood quietly on a force plate in a comfortable side-by-side feet position [33] with their arms relaxed along the trunk. The task consisted in two tests (lasting 60 seconds each) corresponding to two different visual conditions: in the first test, the children were requested to stand upright with eyes open (EO), whereas in the second they were requested to stand upright with eyes closed (EC). Between tests an interval of 2 minutes was allowed. Considering the reported relatively high test-retest reliability of most common posturographic parameters for similar population samples in the tested conditions [34], and the possible effect of fatigue associated with this task [35] only one repetition for each condition was performed.

Force plate signals were used to obtain CoP data in both medio-lateral and antero-posterior directions. Relevant force and torque components were low pass filtered (corner frequency 20 Hz, 8^th^ order elliptical filter, stopband attenuation 80 dB at 30 Hz, attenuation slope 135 dB/octave) and fed to an AD converter (100 samples/s, DAQCard^TM^ – AI-16E-4, by National Instrument Corporation). From the CoP coordinates, the TtB function was extracted following the definition reported in [26]. The function estimates the predicted instance in time (τ) when the instantaneous CoP trajectory (*x_i_*, *y_i_*) would cross the boundary limits, as predicted by a parabolic motion driven by the position (*r_x_*, *r_y_*), velocity (*r_x_*, *r_y_*) and acceleration (*r_x_*, *r_y_*) of the CoP data at time instant t_i_, according to the equations:

$x_{i}(\tau )=r_{x}(t_{i})+r_{x}(t_{i}).\tau +r_{x}(t_{i}).{\frac{\tau }{2}}^{2}$

$y_{i}(\tau )=r_{y}(t_{i})+r_{y}(t_{i}).\tau +r_{y}(t_{i}).{\frac{\tau }{2}}^{2}$

For each age group, the stability boundary was shaped as an ellipse whose axes were determined a priori based on the anthropometric features of the subjects (feet length) and on the distance between the feet. As illustrated in Fig. (**1**) TtB has a characteristic and repeatable behaviour: it switches between minima (when approaching to boundary limits) and maxima (when turning from one direction to another).

From this one-dimensional predictive parameter, it is possible to obtain further parameters that deal with the features of postural control, such as the intervention rate of the postural control system.

Therefore, for each test and for each subject four indicators were extracted from TtB, according to previous works [23, 24]: two spatial parameters – the mean value of the minima detected throughout the trial, and their standard deviation (M_min_, Std_min_), - two temporal ones, the mean value of the temporal distance between successive minima and their standard deviation (M_dist_, Std_dist_). The formulas to calculate the parameters are reported in Table **2**.

In particular, M_min_ could suffer from differences in biomechanical constraints and from the capacity to perceive the limit of stability, and its standard deviation could be dependent on the shape of the CoP trajectory with respect to the bounds of stability. The M_dist_ and its standard deviation parameters could be considered representative of the intervention rate of the postural control system since the inversion of the TtB is a direct consequence of the action exerted by the muscles to move the CoP away from the bounds of stability when TtB is approaching zero.

Statistical analysis was performed on these parameters, to compare the two vision conditions (EO, EC) and the three age groups (Y7, Y9, Y11). Descriptive statistics were calculated, and the Kolmogorov-Smirnov test was done to verify the normal distribution of data. All parameters were analysed through ANOVA with repeated measures with Vision (EO *vs.* EC) and Age (Y7 *vs.* Y9 *vs.* Y11) as factors. To check for age-specific effects on vision, and vision-specific effects on each age group, post-hoc ANOVA analysis was also performed.

## 3 RESULTS

All TtB parameters were affected by Vision and Age, as reported in Table **3**. The statistical results showed that no parameter depended on the interaction Vision x Age, with M_min_ showing p-values at significance limits (p = 0.06). Mean and standard deviation for each parameter are shown in Fig. (**2**).

### 3.1 Effect of Vision on TtB Parameters

To study the effect of vision for each age group, post hoc ANOVA test was done on each group separately for all parameters. The results and the statistical analysis showed significant differences for all parameters in Y7: when the children close their eyes, the two spatial parameters decrease (M_min_ and Std_min_) and the two temporal measures increase (M_dist_, Std_dist_).

Instead, in Y9 a significant effect appeared only in M_min_ and Std_dist_: M_min_ decreased and Std_dist_ increased when the children close their eyes. Finally, no significant difference was shown in Y11. All statistical results are showed in Table **4**.

### 3.2 Effect of Age on TtB parameters

The analysis of the effect of Age on the parameters was done comparing the three age groups in both vision conditions.

As reported in Fig. (**2**), M_min_ increased passing from Y7 to Y9, then preserving its value in Y11, in both EO and EC conditions; this is confirmed by the statistical analysis in Table **5**. Std_min_ showed the highest value in Y9, as compared to Y7 and Y11, with a significant difference only between Y7 and Y9 in both vision conditions. For M_dist_ and Std_dist_ the comparison shows the lowest values in Y11, in both vision conditions. In particular, the statistical analysis shows significant differences between Y7 and Y11 and between Y9 and Y11.

## 4 DISCUSSION AND CONCLUSION

The TtB parameters, calculated for each age group and for the two vision conditions (eyes open and eyes closed), resulted sensitive to both main factors. As such, they provided useful additional information about postural control development in children population that was not detected by the analysis of the traditional postural parameters: the adult-like balance control strategies, that may be associated with a mature development of the integration of vision information, begin to appear at the age of 9 and they look settled at 11.

In a previous study, Schmid *et al.* [14] had shown that the absence of the visual channel leads to a change in postural control strategy, with a transition between 9 and 11 characterized by an increase of the mean amplitude (MA) in Y7 and Y9, and a decrease of MA Romberg Ratios in Y11, as compared to Y7 and Y9.

The measurements extracted from the Time-to-Boundary function, coming from this study seem to integrate those previous findings and to confirm that the variation of these parameters are linked to the variation of the postural stability.

At the age of 7, the decrease of the spatial measure (M_min_), associated with an increase of both temporal measures (M_dist_ Std_dist_), suggests that without the visual channel the control system seems to intervene when the temporal margins to the stability boundaries are lower; moreover, it might come into play less frequently than in the EO condition.

The Time-to-Boundary function and the measurements extracted from it, confirmed the intermittent nature of postural control [36] and highlighted how the postural instability, in absence of the visual channel, depends on a significant change of the CoP motion direction inside the area of stability.

A possible interpretation of the obtained results may be associated with the different nature of the TtB parameters: the spatial ones (M_min_, Std_min_) might be considered as measures of stability margins associated with the controlled variable – the higher their value, the higher the margin from instability conditions for the plant; the temporal ones (M_dist_, Std_dist_) may be directly linked with the controlling mechanisms – a lower value of M_dist_ might be linked with an increased intervention rate of the postural control system needed to obtain the requested stability margins. In absence of vision at age of 7, the decrease of the stability margins (M_min_ lower) and the decrease of the intervention rate (M_dist_ higher) would reflect the still not complete ability of the postural control system to timely intervene to maintain the same margins of stability displayed with eyes open.

Instead, the absence of observed differences for all TtB parameters in Y11 confirmed the hypothesis that, at 11 years, children are able to maintain the same intermittent rhythmical postural control strategy in both visual conditions, and to effectively compensate for the absence of vision. This hypothesis was confirmed by the absence of a significant effect of vision, for all TtB measures, at 11, in the same way as the results obtained in a young adult population sample [37].

Age of 9 resulted critical: in absence of visual channel, despite the decrease of the stability margins (M_min_ lower), the postural control system maintains the same intervention rate of the eyes open condition (M_dist_ not affected), but with higher variability (Std_dist_ higher). The presence of an increase in the variability of the intervention rate may reflect that the absence of vision, at this age, might have a residual effect on the regularity of the postural control system, which still leads to reduced margins of stability.

Looking at the effect of having the eyes closed, interpretation of results from the studies based on classical parameters is disputed: Riach and Hayes (1987) have shown no difference between eyes open and eyes closed [9]. They hypothesized that children use visual information to control balance in a manner different from adults until after the age of 7 years. Conversely, Wolff *et al.* (1998) suggested that children, even at a younger age, are more unstable in absence of information coming from the visual channel [38]. The previous research conducted using classical parameters proposed that children between 7 and 9 start to put in action a more accurate and restrained control strategy [9] and Peterson *et al.*. (2006) suggested that the mature postural responses emerge at around 12 [11].

We believe that the results obtained from the present study therefore, substantiate the hypothesis that the age of 9 is a critical point in the development of postural control. The numerical results also highlight a subtler change: they suggest that in the same way as at 11, at 9 the children are able to maintain the balance effectively predicting the temporal limits of stability, but with a higher variability. This is confirmed by the increase of M_min_, as compared to 7, and by the increase of Std_min_ as compared to 11.

Therefore, we can speculate that at 9, children “explore” their limits of stability and that at 11 they have completed the maturation of postural control with a stable management of spatial-temporal information. This hypothesis is confirmed by the decrease of both M_dist_ and Std_dist_ measures at 11, as compared to 9.

We can conclude that the study of TtB measurements provides interesting and additional information to predict the development of postural control in children population.

Further studies will be needed to better explore the mechanisms that participate in postural control development. It could be of interest to study how the measurements extracted from the Time-to-Boundary function can provide information about the relationship between cognitive development and postural control. Furthermore, the TtB may as well provide additional information about the postural control in children with disorders [39] (*i.e.* Children with visual channel deficits and/or vestibular deficit). A complete study could associate the recording of the EMG signals to estimate possible modifications of muscular activation in upright stance [40] in different age groups and vision conditions. Rehabilitation or training systems able to leverage on the evaluation of postural function [41] based on the analysis of predictive parameters could then be developed.

## Figures and Tables

**Fig. (1):**
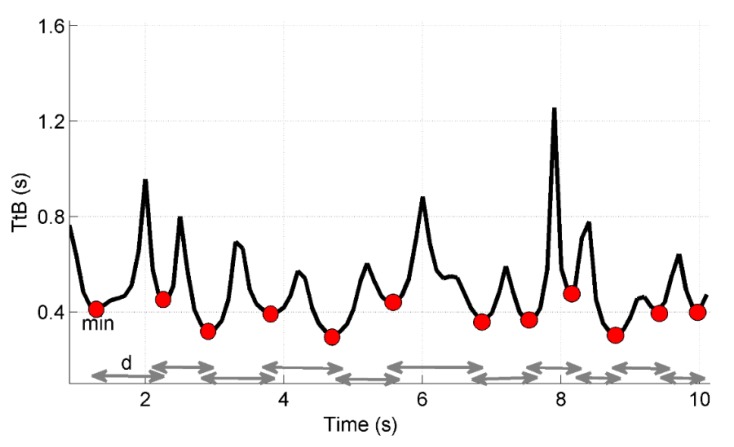
Sample of a Time-to-Boundary function from recorded data: red spots indicate the minima of the function (*min*), while grey arrows represent the distances between successive minima (*d*).

**Fig. (2):**
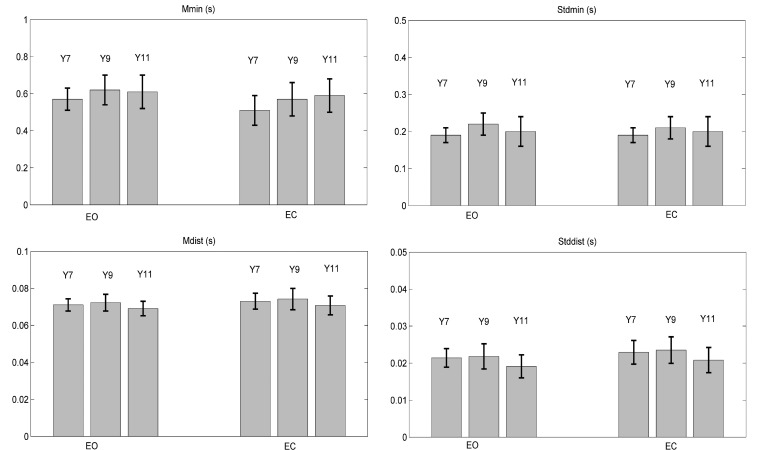
Mean ± standard deviation of all the TtB parameters (M_min_, Std_min_, M_dist_, Std_dist_), for each age group (Y7, Y9, Y11) and for both vision conditions (eyes open - EO, eyes closed - EC).

**Table 1:** Population anthropometric data (group mean ± standard deviation).

**Age Group**	**Y7**	**Y9**	**Y11**
**N**	41	38	28
**Age (yrs)**	Range 6.5-7.5	Range 8.0-9.8	Range 10.5-12.0
**Height (m)**	1.22 ± 0.06	1.34 ± 0.07	1.46 ± 0.06
**Weight (kg)**	25.3 ± 4.7	32.5 ± 7.1	43.1 ± 8.7
**Feet length (m)**	0.17± 0.01	0.19± 0.02	0.20 ± 0.01

**Table 2:** TtB parameters.

*M_min_* = *E*{*min*}	*M_dist_* = *E*{*d*}
*Std_min_* = [*E*{*min*}^2^ - *M_min_*^2^]^½^	*Std_dist_* = [*E*{*d*}^2^ - *M_dist_*^2^]^½^

*E*{} denotes the expected value of the variable *min* is the array of minimum values of the TtB function. *d* is the array of temporal distances between successive minima of the TtB function.

**Table 3:** 2-way ANOVA results: F-values and p-values for all the TtB parameters (M_min_, Std_min_, M_dist_, Std_dist_) considering main factors (Vision and Age) and their interaction (Vision x Age).

	**VISION**	**AGE**		**VISION x AGE**
**M_min_**	F=39.12; (p <0.001)		F=6.61; (p <0.01)	F=2.74; (p= 0.06)
**Std_min_**	F=4.24; (p =0.04)		F=7.97; (p <0.01)	F=0.03; (p =0.97)
**M_dist_**	F=22.82;(p <0.001)		F=5.73; (p <0.01)	F=0.07; (p =0.93)
**Std_dist_**	F=30.18;(p <0.001)		F=5.88; (p <0.01)	F=0.06; (p =0.93)

**Table 4:** Post-hoc ANOVA: F-values and p-values of Vision factor (EO *vs.* EC) for each age group (Y7, Y9, Y11). n.s -no significant.

	**Y7- EO *vs.* EC**	**Y9 – EO *vs.* EC**	**Y11- EO *vs.* EC**
**M_min_**	F=10.69; (p = 0.01)	F=4.49; (p =0.03)	n.s.
**Std_min_**	F=3.98 (p=0.05)	n.s.	n.s.
**M_dist_**	F=5.43; (p =0.02)	n.s.	n.s.
**Std_dist_**	F=5.16; (p =0.02)	F=4.26; (p =0.04)	n.s.

**Table 5:** Post-hoc ANOVA: F-values and p-values of Age factor, for each visual condition (EO, EC). The analysis was done comparing the groups in pairs (Y7 vs. Y9, Y7 vs. Y11, Y9 vs. Y11). n.s.- no significant.

	Y7 vs. Y9		Y7 vs. Y11		Y9 vs. Y11	
	EO	EC	EO	EC	EO	EC
M_min_	F=8.39; (p =0.004)	F=9.14; (p =0.003)	F=4.79; (p=0.03)	F=13.57; (p <0.001)	n.s.	n.s.
Std_min_	F=11.24; (p =0.001)	F=12.4; (p =0.007)	n.s.	n.s.	n.s.	n.s.
M_dist_	n.s.	n.s.	F=5.08; (p =0.02)	F=4.02; (p=0.04)	F=10,6; (p=0.001)	F=6.47; (p=0.01)
Std_dist_	F=0.35; (p =0.55)	F=0.66; (p =0.66)	F=11.57; (p =0.001)	F=7.03; (p=0.01)	F=10.94; (p =0.001)	F=9.76; (p=0.001)
